# Application of the ADAPT Framework to Contextualize a Participatory Learning and Action Community Intervention for the Prevention and Control of Type 2 Diabetes Mellitus in Urban and Rural Settings in Afghanistan and Pakistan: Protocol for Intervention Adaptation

**DOI:** 10.2196/71602

**Published:** 2026-03-27

**Authors:** Amber Tahir, Noor Sanauddin, Sara Imtiaz, Nadia Khaleeq, Mariam AbdeAli, Asima Khan, Farrukh Ahmed, Hannah Maria Jennings, Rubia Zafar, Khalid Rehman, Abdul Rahman Shahab, Saima Afaq

**Affiliations:** 1 Health Promotion Foundation, Karachi, Pakistan; 2 Department of Sociology, University of Peshawar, Peshawar, Pakistan; 3 Institute of Public Health and Social Sciences, Khyber Medical University, Peshawar, Pakistan; 4 Department of Health Sciences, Hull York Medical School, University of York, Heslington, United Kingdom; 5 HealthNet TPO, Afghanistan Country Program, Kabul, Afghanistan; 6 Department of Health Sciences, University of York, Seebohm Rowntree Building, Heslington, York, YO10 5DD, United Kingdom, 44 (01904) 323977

**Keywords:** implementation, co-design, behavior change, noncommunicable disease, contextual, public health, mixed method, low- and middle-income countries, participatory research, formative study, Southeast Asia

## Abstract

**Background:**

Type 2 diabetes mellitus (T2DM) is a critical global health issue, especially in low- and middle-income countries like Afghanistan and Pakistan, where many cases remain undiagnosed and specialized care is costly. Community Health Participatory Research, which actively involves community members in addressing health issues, is increasingly recognized as an effective approach to deliver sustainable and culturally relevant health solutions. Participatory Learning and Action (PLA), a method of Community Health Participatory Research, was implemented in the D-Magic (Diabetes Mellitus: Action Through Community Groups or mHealth Information for Better Control) trial in Bangladesh that empowered communities to identify challenges and develop locally relevant strategies to prevent and control T2DM.

**Objective:**

This study aims to adapt the PLA-based D-Magic intervention for the prevention and control of T2DM in rural and urban communities of Afghanistan and Pakistan.

**Methods:**

This trial, titled “Engagement of Community Through Participatory Learning and Action for Control and Prevention of Type 2 Diabetes and Its Risk Factors” (EMPOWER-D), will systematically adapt the D-Magic intervention using the ADAPT guidance framework. The study will be conducted across 3 sites: rural Afghanistan (Kabul), rural Pakistan (Peshawar and Swabi), and urban Pakistan (Karachi). Stakeholders and community members will be involved to ensure cultural relevance and authenticity. The process includes identifying barriers and facilitators to the implementation of community-based interventions through a qualitative study and a scoping review, co-designing intervention materials, and piloting for feasibility and effectiveness.

**Results:**

EMPOWER-D was funded and launched in November 2022 by the United Kingdom’s National Institute for Health and Care Research (NIHR203248). Following a year-long development phase, the adaptation protocol was finalized, and a mock run of the interviews was conducted. Recruitment and qualitative data collection, including in-depth interviews and focus group discussions, began in July 2024 and have been completed in Peshawar, Swabi, and Karachi. As of January 2025, data collection has commenced in Afghanistan. Results are anticipated to be published in mid-2026 following the completion of data analysis.

**Conclusions:**

The EMPOWER-D adaptation will align the PLA-based intervention with the local sociocultural context, ensuring health equity and participation. Building on the D-Magic trial’s success, this adaptation will ensure the intervention’s effectiveness and cost-effectiveness in preventing and managing T2DM, not only in Afghanistan and Pakistan but also in other low- and middle-income countries.

## Introduction

### Background

Type 2 diabetes mellitus (T2DM) is a major global health issue, with 463 million adults affected globally, 80% of whom are living in low- and middle-income countries (LMICs) [1]. This health crisis is further exacerbated by conflicts, political instability, and natural disasters that disproportionately affect LMICs [2,3] such as Afghanistan and Pakistan. Afghanistan faces significant challenges, with an estimated 11.07% of adults having T2DM and 10.32% having impaired hyperglycemia [4]. Similarly, in Pakistan, the world’s fifth most populous country, 26.7% of adults (approximately 33 million people) have T2DM [5]. Additionally, many cases of T2DM in both countries are undiagnosed, worsening the health and economic burden of the condition.

Environmental changes, epidemiological transition, genetic predisposition, and behavioral factors such as diet and physical inactivity contribute to the rising incidence of T2DM in LMICs, in countries such as Afghanistan and Pakistan [6,7]. The disease impacts physical health, imposing significant emotional, social, and economic strains on individuals and communities [8]. T2DM resulted in 75.3 million (95% uncertainty interval 63.5‐90.2) global disability-adjusted life years in 2021 and was responsible for 2.6% (95% uncertainty interval 2.3%‐2.9%) of total global disability-adjusted life years [9].

Recent advances in health care have suggested addressing T2DM through specialized care, including a multidisciplinary approach to address diabetes-related complications [10]. However, this approach places a continuous and significant burden on individuals due to high out-of-pocket costs [11,12] for frequent medical visits, expensive medications, and diagnostic tests. Furthermore, the focus is usually placed on the management rather than the prevention of the disease, which further adds strain on already stretched health care systems, making diabetes management less accessible for many. In contrast, community-based participatory approaches are becoming increasingly popular because of their effectiveness in improving diabetes outcomes [13]. Moreover, community-based participatory approaches to health address the underlying causes of the disease and help in the prevention as well as the management of the health issue [14].

Community Health Participatory Research (CHPR) is an inclusive approach that actively involves community members in identifying areas for intervention, defining the problem, and generating potential solutions [15]. These approaches prioritize collaboration, equity, and the cocreation of knowledge to produce more relevant and effective interventions [16]. One CHPR approach is Participatory Learning and Action (PLA). PLA was inspired by Paulo Freire’s philosophy, where community members actively participate in learning about, defining, and addressing their health issues through facilitated sessions and activities [17]. PLA empowers marginalized communities to identify and solve their problems through a cycle of problem identification, solution planning, implementation, and reflection rather than relying on top-down strategies [18]. PLA has been systematized into a group-based intervention and has proven effective in improving neonatal and child health [19,20]. More recently, this approach was adapted to address diabetes in rural Bangladesh through the D-Magic (Diabetes Mellitus: Action Through Community Groups or mHealth Information for Better Control) trial [21]. The PLA intervention that was tested led to a 48% reduction in the prevalence of T2DM compared to the control group (305/3757, 8% vs 493/3821, 13%, respectively), with an adjusted odds ratio of 0.52 (95% CI 0.38‐0.71; *P*<.001). Additionally, the intervention increased awareness of T2DM prevention and motivated healthy dietary changes [21]. The D-Magic trial successfully used a participatory approach, organizing communities into small groups that held monthly meetings led by a lay facilitator. These groups followed a 4-phase PLA cycle to prevent and control T2DM, identifying barriers to healthy behavior and developing and implementing context-specific strategies, such as raising awareness, group exercise, income generation, and kitchen gardening [21].

The EMPOWER-D (Engagement of Community Through Participatory Learning and Action for Control and Prevention of Type 2 Diabetes and Its Risk Factors) trial is grounded in the conviction that PLA can empower communities to take ownership of diabetes prevention and control efforts. By organizing communities for collective action, PLA aims to increase awareness and motivation for healthy behaviors, thereby enabling individuals to make informed choices about their health. As a result, individuals are expected to gain confidence and motivation to seek health care services and adopt healthier habits. Ultimately, this approach is anticipated to lead to the development of a self-sustainable model for the prevention and control of diabetes, which can also have a positive impact on the prevention and control of other noncommunicable diseases (NCDs) in the region.

The theory of change logic model for our PLA intervention in EMPOWER-D (Multimedia Appendix 1) illustrates the hypothesized pathways through which participatory group activities are expected to lead to improved diabetes prevention and control in fragile LMIC contexts. It was developed by the research team, based on the findings from the D-Magic process evaluation and the context of Pakistan and Afghanistan. This model underpins our adaptation approach and will guide both implementation and evaluation.

Participatory interventions like PLA are highly context-sensitive, requiring adaptation to achieve sustainable outcomes when implemented in new settings or with different populations [22]. Despite its effectiveness and greater acceptability among the people, evidence on how PLA interventions like D-Magic can be adapted for fragile, crisis-affected, and socially diverse LMICs remains scarce. This is particularly relevant in countries with high T2DM burdens, such as Afghanistan and Pakistan, where direct replication could overlook important contextual distinctions [23,24]. These include variations in health care system capacity, cultural differences, gender-segregated communities, urban-rural disparities, and the added burden of conflict and political instability [25]. In Afghanistan, decades of conflict have overshadowed public health efforts, including addressing NCDs like T2DM, likely resulting in underreported prevalence [26]. In Pakistan, although the situation is relatively stable, the country has faced repercussions due to its proximity to unstable regions and a rapidly changing political environment, which adds to the issues of rapid urbanization and health care disparities, particularly between urban and rural areas, and for women in conservative rural regions [27]. These challenges contribute to rising T2DM rates and a fragmented health care system, with private urban centers offering better care than underfunded public clinics [28].

These differences shape how communities perceive health, engage with interventions, and mobilize collective actions. Thus, this study does not aim for simple transferability of D-Magic but for systematic adaptation that will generate new evidence on how participatory approaches can be tailored for fragile, gender-segregated, and highly diverse LMIC contexts.

### Aims and Objectives

This work aims to provide the following:

1. Empirical insights into barriers and facilitators of community-based diabetes prevention in crisis-affected and urbanizing LMICs
2. Methodological advances by combining scoping reviews, qualitative inquiry, and co-design
3. Practical frameworks for incorporating participatory diabetes interventions in health systems where trust, equity, and access are major challenges.

## Methods

### Ethical Considerations

Ethical approval for this study has been granted by the ethics review committees of the Aga Khan University, Karachi, Pakistan (2024-9340-28927); Khyber Medical University, Peshawar, Pakistan (KMU/IPHSS/Ethics/2023/EO/0136); HealthNetTPO, Kabul, Afghanistan (A-12-24-464); and the National Bioethics Committee (NBC-R-1070). Researchers will approach identified participants and inform them about the research verbally and through a participant information sheet in their local language (Pashto, Dari, and Urdu). Participants will be encouraged to ask about the research before deciding whether they would like to participate. For those who consent to participate and provide a written consent, a time and date for an in-depth interview (IDI) or focus group discussion (FGD) will be arranged. Interviews will be conducted in a comfortable and confidential environment to ensure participants’ privacy. Participants will be assigned unique identification codes, and no personal identifiers will be included in transcripts or analysis files. Audio recordings will be stored in password-protected, encrypted folders and deleted after transcription. Deidentified transcripts will be used for analysis. All electronic data will be stored on secure, password-protected devices, and hard copies (eg, consent forms) will be kept in locked cabinets. Only authorized research team members will have access to the data, and findings will be reported in aggregate to protect participant identity. Participants will receive a monetary incentive to cover their travel expenses and compensate them for their time spent participating in the interview.

The full version of the EMPOWER-D trial is registered with ClinicalTrials.gov (NCT07350694 for rural Afghanistan, registered January 20, 2026, NCT06561126 for rural Pakistan, registered August 23, 2024, and NCT06570057 for urban Pakistan, registered August 26, 2024).

### Study Overview

This study is part of the research program EMPOWER-D. The EMPOWER-D trial, a project of Centre for Improving Mental and Physical Health Together (C4IMPACT), was launched to specifically address the prevention and control of T2DM in rural and urban communities of Afghanistan and Pakistan.

### Conceptual Framework

We will utilize the ADAPT guidance [22] to adapt the intervention systematically. The ADAPT guidance framework emphasizes the importance of involving stakeholders and community members in the adaptation process to ensure that the adapted evidence-based intervention (EBI) is culturally appropriate and contextually relevant.

**Figure 1.**
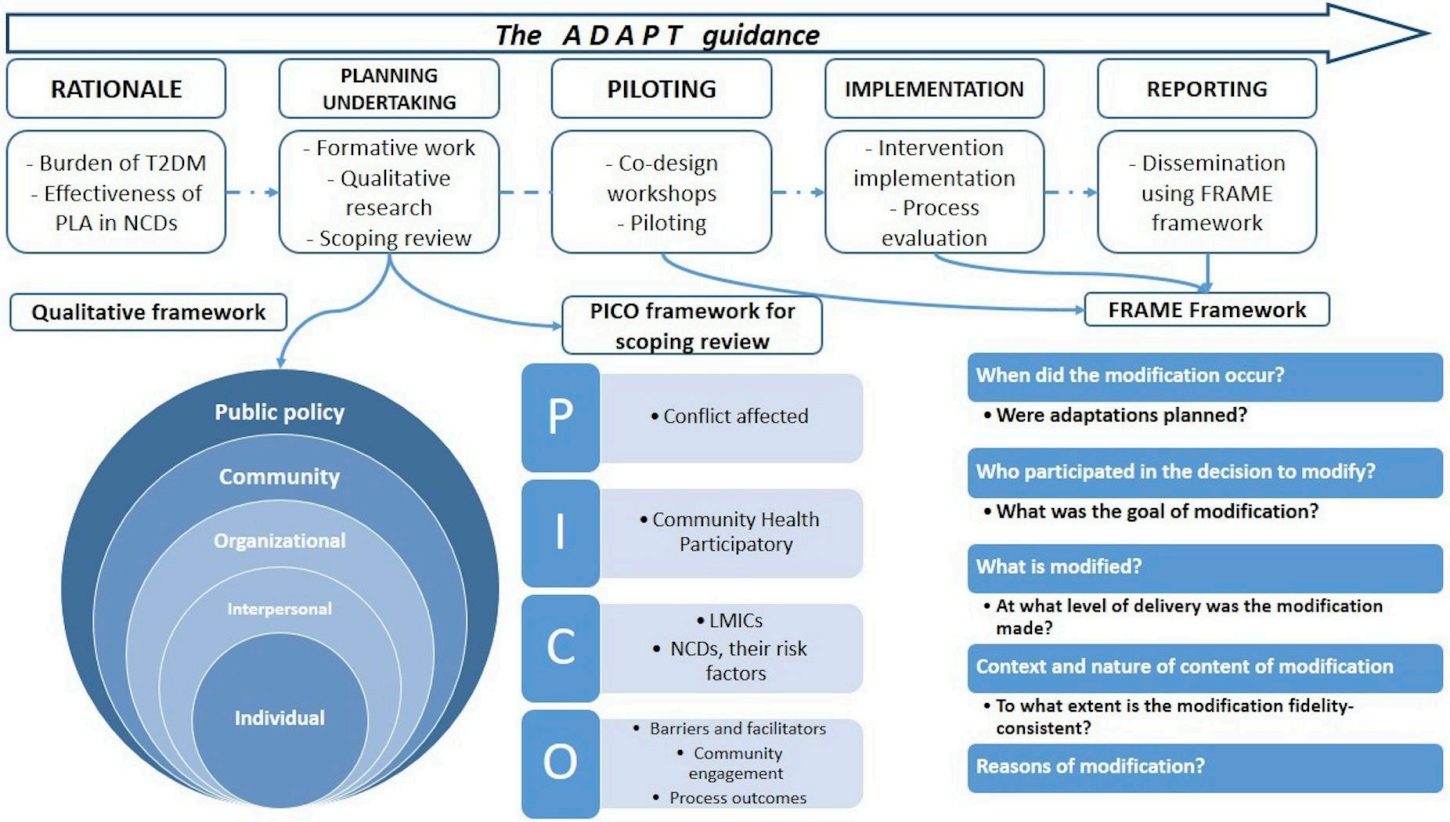
Conceptual framework of the process of adaptation in EMPOWER-D (Engagement of Community Through Participatory Learning and Action for Control and Prevention of Type 2 Diabetes and Its Risk Factors). FRAME: Framework for Reporting Adaptations and Modifications in Evidence-Based Interventions; LMICs: low- and middle-income countries; NCDs: noncommunicable diseases; PLA: Participatory Learning and Action; T2DM: type 2 diabetes mellitus; PICO: Population, Intervention, Comparison, Outcome.

The framework comprises 5 steps to guide the adaptation process (Figure 1). This study will adapt the D-Magic intervention by carrying out three main activities: (1) identifying the barriers and facilitators in the implementation of community-based interventions (CBIs) and their contextual appropriateness through a qualitative study (using the socioecological model (SEM) [29]) with local stakeholders and a scoping review of the published literature, (2) co-designing the intervention materials, and (3) piloting the adapted materials for feasibility and effectiveness.

The adaptation of intervention materials across the 3 sites (rural Afghanistan, rural Pakistan, and urban Pakistan) will vary according to each context, but the process of adaptation will be the same. The process and findings will be documented using the FRAME (Framework for Reporting Adaptations and Modifications in Evidence-based Interventions) framework [30] (Figure 1), detailing the changes made, the reasons for these changes, and the implementation procedures. The outcomes and implications of the study will be recorded and reported, with recommendations for future research and practice.

### Phase 1: Identifying Barriers and Facilitators to the Implementation of CBIs

#### Step 1A: Qualitative Study

##### Overview

We will conduct a qualitative study to explore the understanding of T2DM in rural and urban communities of Pakistan and Afghanistan, the barriers and facilitators to the uptake and maintenance of healthy behaviors related to T2DM, and their willingness to engage in CBIs. We selected a qualitative approach because health-related behaviors are deeply shaped by cultural beliefs, gender roles, and community structures that cannot be adequately captured through quantitative methods. Qualitative inquiry will also allow us to capture the lived experiences and perspectives of diverse community stakeholders, contributing to context-rich insights that are essential for tailoring participatory interventions to fragile and heterogeneous settings. The SEM emphasizes the interaction of individual, interpersonal, organizational, and community-level factors in shaping health outcomes [28]. The SEM was preferred as it offers a comprehensive framework to explore diabetes-related barriers and facilitators across multiple levels, which is essential for designing and adapting CBIs.

##### Study Settings

In Afghanistan, the feasibility trial will be conducted in Farza and Dehsabz, Kabul district. The trial will mainly focus on the rural population in the Kabul district, which is populated mainly by people from lower socioeconomic backgrounds. Protracted conflicts and political instability have greatly affected the health care system in the district.

In Khyber Pakhtunkhwa, the trial will be conducted in the districts of Peshawar and Swabi. Peshawar, the densely populated capital, has 4.7 million [31] residents and offers comprehensive health care through public and private sectors. Swabi, with 1.9 million people [31], provides primary and secondary care through government facilities supplemented by private health care. Both predominantly rural districts, inhabited by Pashtuns, faced challenges of poverty, limited infrastructure, under-resourced education, and conflicts, resulting in reduced economic growth and development for the past decade.

For the urban feasibility trial in Pakistan, the District Central of Karachi, Sindh, has been selected. With a population of approximately 3.8 million [32], it is one of the most densely populated areas in Karachi, which itself houses 17 to 20 million people. The district’s diverse population includes various ethnic groups, and livelihoods range from white-collar jobs to informal labor. Education access is uneven, with significant disparities between elite private schools and underfunded public institutions. Karachi’s extensive health care system includes public, private, and nonprofit sectors, but it faces overcrowding and unequal access, particularly in public hospitals.

##### Participants and Recruitment

Participants will be recruited through purposive sampling from the 3 study sites. The inclusion criteria are adult (over 18 y of age) male and female participants residing within the geographical boundaries of the selected sites. Participants must be able to communicate effectively in either Pashto, Urdu, or Dari (or any other local language) for the interviews and be willing to share their experiences and opinions openly. Participants who have already participated in similar interviews or focus groups for the same study will be excluded to avoid redundancy and bias. Maximum variation will be ensured by recruiting participants with different demographic characteristics (gender, socioeconomic status, geographical spread, age, ethnicity, etc). A team of male and female researchers will go to the field to recruit participants through their professional contacts and snowballing. In addition, community advisory panels (CAPs) were formed at all study sites, comprising members of the community, such as religious leaders, community elders, health care professionals, individuals living with T2DM, and caregivers. These CAP members will also be requested to help identify potential participants.

##### Sampling

Five categories of stakeholders, guided by the SEM, will be recruited to explore the views and perspectives of people living with T2DM and people with unknown T2DM status, carers, influential people, and health care providers (Table 1).

Specifically, we will recruit the following:

1. Carers of people with T2DM, adults who are the primary caregivers for someone living with T2DM, are to provide insights into household dynamics and support systems.
2. People with unknown T2DM status, adults who have never been diagnosed with T2DM, and who do not self-identify as having the condition. This group may include individuals with undiagnosed T2DM, as they are unaware of their glycemic status at the time of recruitment, to capture perceptions, beliefs, and practices around prevention, lifestyle, and risk awareness.
3. Influential people, Imams (religious leaders), community elders, teachers, elected representatives, etc, are engaged, given their influence on collective norms and decision-making.
4. A range of health care providers, including doctors, nurses, pharmacists, and community health workers, who treat and care for people with T2DM will be interviewed to reflect organizational and health system perspectives.
5. People with T2DM, adults living with T2DM (who have had a medical diagnosis), to capture both lived experiences of disease management and perceptions of risk and prevention.

As shown in Table 1, a total of 60 IDIs and 16 FGDs with the aforementioned 5 categories of participants will be conducted at each site. Efforts will be made to recruit an equal number of male and female participants across the study sites. In line with qualitative research principles, our sampling strategy is guided by the principle of thematic saturation. The planned sample size is sufficiently large to capture a diverse range of perspectives across major stakeholder groups while allowing flexibility to adjust numbers until saturation is reached.

**Table 1.** Participants’ categories and sample size for in-depth interviews (IDIs) and focus group discussions (FGDs).

Participants’ category and interview type	Sample size			
	Rural Pakistan (Peshawar and Swabi)	Urban Pakistan (Karachi)	Rural Afghanistan (Kabul)	Total sample
People living with T2DM^a^				
IDIs	4	4	4	12
FGDs	4	2	2	8
People with unknown T2DM status				
IDIs	4	4	4	12
FGDs	4	2	2	8
Carers of people with T2DM				
IDIs	4	4	4	12
Health care providers				
IDIs	4	4	4	12
Community influentials				
IDIs	4	4	4	12

a T2DM: type 2 diabetes mellitus.

Data saturation will be evaluated through an iterative process of concurrent data collection and analysis. After each round of interviews and FGDs, the research team will review the transcripts to identify emerging codes and themes. Saturation will be considered reached when successive interviews no longer yield new codes or meaningfully expand existing categories. To enhance rigor, at least 2 researchers will independently assess saturation and maintain a saturation grid to document the point at which no new information emerges. This approach ensures a clear, evidence-based justification for the adequacy of the sample and the credibility of the findings.

##### Data Collection

The IDIs and FGDs will be conducted by a team of male and female research assistants trained in qualitative research methods. An experienced research team member will supervise the fieldwork. The research team will develop topic guides based on the research objectives and the conceptual framework. FGDs and IDIs are valuable research techniques, and by triangulating these methods and participants, we aim to obtain rich information from various sources. The data collection techniques are described below:

1. In-depth interviews: Interviews will be conducted by research assistants with support from a notetaker and are expected to last 45 to 60 minutes. IDIs will be conducted with all 5 categories of participants in a peaceful place convenient to them. With participants’ consent, interviews will be recorded using a digital audio recorder (Multimedia Appendices 2-6).
2. Focus group discussions: FGDs will involve people living with T2DM and people with unknown T2DM status to foster interactive discussions and gain insights into community beliefs and experiences. Separate FGDs will be held for males and females and those with T2DM and with unknown T2DM status to capture diverse perspectives. Discussions will occur at convenient locations, such as local community centers or public spaces, and will be facilitated by a moderator and a notetaker and are expected to last 60 to 90 minutes. With participants’ consent, FGDs will be audio-recorded (MultimediaAppendices 7  8).

##### Data Analysis

The recordings of IDIs and FGDs will be transcribed and translated into English. Transcripts and field notes will be uploaded onto a secure server, which is accessible to the research team only. Sections of transcriptions and translations will be checked for accuracy and quality by a senior research team member who is not directly involved in the data collection and transcription and who is bilingual.

The transcripts will be carefully read to reflect participants’ articulated meanings and experiences. Initially, open and inductive coding of five transcripts will be conducted to create a list of recurring codes within the data. Based on these initial codes, a coding framework will be prepared in a Microsoft Excel worksheet. After that, the transcripts will be deductively coded and charted into the coding framework, which will be flexible enough to accommodate more codes during the charting process [33]. Next, codes will be merged into categories and themes [34]. The themes identified deductively will be inductively organized using SEM [29] to provide a broader context for the findings. Finally, summaries of all the themes will be prepared to address the research questions.

To ensure the quality of the analysis, data sources (IDIs and FGDs) will be triangulated to confirm the validity and consistency of the findings. Throughout the analysis process, researchers will be encouraged to reflect on their positionalities and place in the study and discuss coding schemes within the team (reflexivity). Peer debriefing will involve discussions with colleagues or experts to review and critique the coding and thematic development process, enhancing the credibility of the analysis. Oversight by experienced researchers will be conducted to review the coding and thematic development for consistency and reliability.

The data will be analyzed separately for Pakistan’s urban and rural areas and Afghanistan’s rural areas, resulting in 3 distinct reports. A combined summary of these reports will then be prepared to inform the adaptation of the PLA-based intervention.

### Step 1B: Scoping Review

#### Overview

We will conduct a scoping review to map evidence on CHPR interventions for NCDs in crisis-affected LMICs. Because the evidence on PLA for the prevention of NCDs and control in crisis-affected LMICs is still limited and poorly characterized, a scoping review is more appropriate than a full systematic review. This approach allows us to maintain methodological rigor while synthesizing a wide range of study designs and contexts that might otherwise remain fragmented. It will also highlight critical insights into how participatory approaches have been implemented and sustained or perhaps failed in crisis-affected settings. The findings will complement our qualitative inquiry by highlighting transferable elements and best practices, while also identifying context-specific gaps that require tailored adaptation in Afghanistan and Pakistan.

The objective of this review is to identify, describe, and characterize community-based participation interventions and the process outcomes of CHPR interventions; explore the barriers and facilitators to community involvement in such interventions; and provide insights into how participatory approaches have been implemented and sustained in fragile settings.

#### Guidelines and Framework

The review will follow the Joanna Briggs Institute guidelines and comply with the Arksey and O’Malley Framework [35], and the findings will be reported as per the PRISMA-ScR (Preferred Reporting Items for Systematic Review and Meta-Analysis Extension for Scoping Reviews) checklist [36]. The scoping review has been prospectively registered at Open Science Framework [37], providing detailed information on screening, extraction, and outcomes.

#### Eligibility Criteria

Inclusion criteria will comprise all original papers published in English on CHPR interventions and NCDs in crisis-affected LMICs, focusing on the adult population (age 18 y or more). In the case of mixed-aged populations (those below and above 18 y), pertinent information specific to the eligible population will be extracted, where possible. Studies with crisis-affected populations, including refugee and internally displaced populations, as specific groups or subsets within the target population, will also be considered and included for data extraction.

Studies will be excluded if they have a pharmacological or genetic focus, target infectious, communicable diseases, focus on domestic, sexual, or gender-based violence, or are conducted in a noncommunity or clinical setting.

#### Databases and Screening

A total of 4 databases will be searched: PubMed, Web of Science, Scopus, and Google Scholar. The search strategy will focus on 4 key areas, such as community participation, NCDs, LMIC, and crisis-affected settings, and will be adapted for each database. The reference list of the included studies will be assessed for additional sources.

**Figure 2.**
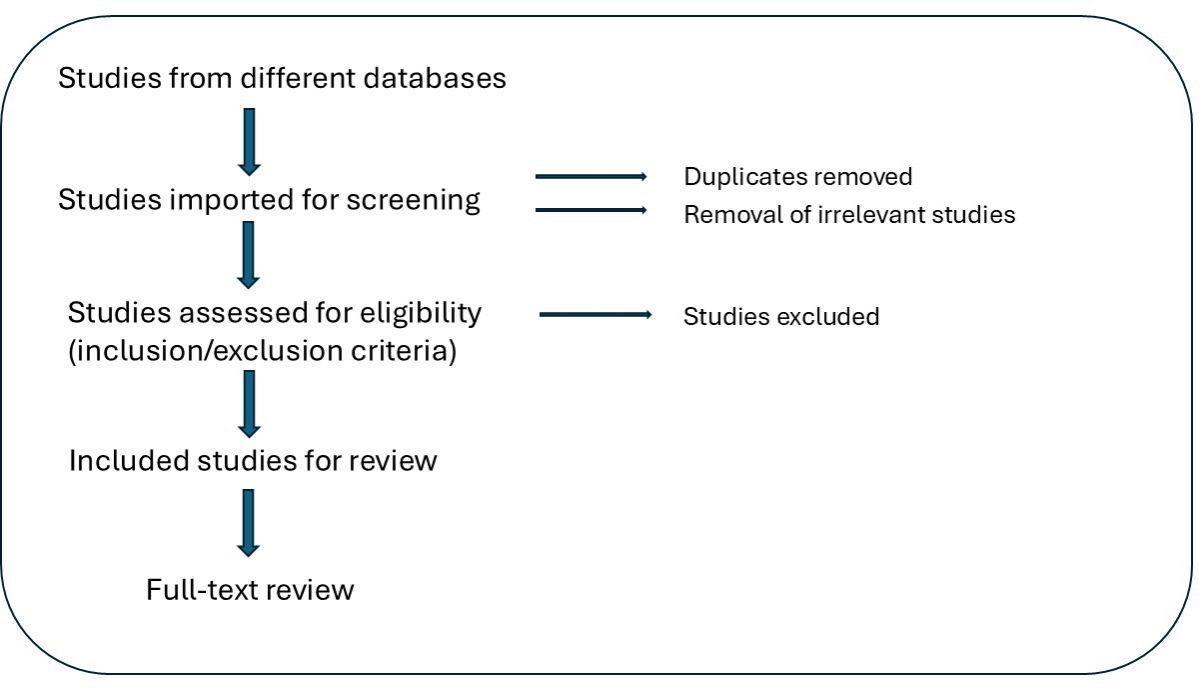
Flowchart of the screening strategy.

All retrieved records will be imported into Covidence for de-duplications and screening. Two reviewers will independently review screen titles, abstracts, and full text against eligibility criteria as presented in Figure 2.

#### Data Extraction and Charting

A standardized data extraction template will be developed, piloted, and refined before use. Two independent reviewers will independently extract data on predefined characteristics as per study objectives. Disagreements at any stage of review will be resolved through discussion or by consulting a third reviewer.

Data will be synthesized narratively to highlight patterns across studies, identify transferable elements and best practices, and outline evidence gaps. As consistent with the scoping review, no formal risk of bias assessment will be undertaken for included studies. Although this is appropriate for the review type, it represents a methodological limitation that will be acknowledged in interpreting the findings.

#### Linking Formative Findings

The findings of the qualitative study and the scoping review will be discussed with the CAP members, including community representatives, to validate and increase the relevance of the findings to the local contexts. Furthermore, these findings will not remain at a descriptive level but will be systematically fed into the adaptation process. Subsequently, a draft of major components and materials of the PLA intervention will be developed and shared with stakeholders in co-design workshops for their input and consensus.

### Phase 2: Co-Designing the Intervention Materials

Co-design methods will be employed to culturally, relevantly, and context-specifically adapt PLA interventions for the prevention and control of T2DM, using the findings from qualitative and scoping reviews [38]. Relevant stakeholders, including the research team, representatives of the local communities, health care providers, individuals with T2DM or those at risk, and graphic designers (for the preparation of materials), will be engaged in the co-design process to foster a collaborative approach to creating and refining strategies that are effective, sustainable, and acceptable to those who will implement and benefit from them. Co-design methods will ensure that the intervention is grounded in the sociocultural context and the lived experiences of the community, leading to more comprehensive and long-term solutions.

#### Process of Integration Through Co-Design

Through a series of co-design workshops (2‐4 per site), with the stakeholders mentioned above, the PLA intervention will be adapted for each site. The details of the workshops will be decided closer to the time; we will also allow for flexibility. However, each workshop will combine findings from formative qualitative research with insights drawn from the scoping review, highlighting transferable lessons and best practices as well as gaps where evidence is weak. This deliberate integration of global evidence with local perspectives will ensure that the adaptation process is both evidence-informed and context-responsive, allowing the formative work (scoping review and qualitative study) to inform the intervention design iteratively and in real time.

To account for contextual diversity, the data collected from the 3 study sites (urban Pakistan, rural Pakistan, and rural Afghanistan) will undergo separate analysis, and distinct co-design workshops will be conducted in each site. This approach ensures that although the overall adaptation framework is consistent, the outputs will be site-specific PLA interventions, tailored to the unique needs and realities of each context, instead of a one-size-fits-all approach. Scoping review findings will be systematically mapped onto key decision points in the workshops (eg, content gaps, delivery mechanisms, and best practices), enabling participants to cross-validate global evidence with local insights. This structured process of triangulation and documentation will help prevent fragmentation and instead generate cohesive, contextually adaptive intervention packages.

The workshops are likely to look as follows:

- Workshop 1: In the first workshop, participants will be introduced to the intervention and materials, with an overview of the EMPOWER-D project, PLA, the target population, key components, and the goals of the intervention. Formative research findings will be shared, highlighting community needs, preferences, and challenges related to T2DM prevention and control. Group activities and discussions will then identify areas of the intervention materials that need review or adaptation.
- Workshop 2: In the second workshop, specific sections of the materials will be reviewed in detail—this is likely to be done through group work. Groups will assess content and context and propose changes or new aspects. The groups will present their findings, and the discussion will focus on reaching a consensus on the necessary changes. These changes will be prioritized based on feasibility, impact, and relevance to the local context.
- Workshop 3: The third workshop will review the changes made to the intervention materials. Revised materials will be presented, and participants will review and provide feedback on the effectiveness and clarity of the revisions. Any final adjustments will be discussed and agreed upon.
- Workshop 4: The final workshop will focus on practical aspects of the intervention, such as meeting places, session frequencies, and group sizes. The discussion will consider logistical factors, such as accessibility, timing, cultural appropriateness, and translation of materials in the local language. Participants will collaborate to finalize the format of the intervention, document decisions, and review the implementation plan and timeline.

These workshops will include approximately 10 to 12 participants representing different stakeholders and take place 1 week apart, leaving enough time for the research team to prepare the agenda, required documents, and activity plan for the next workshop.

#### Managing Contextual Challenges and Trade-Offs

Given the fragile and diverse contexts in which this study will be conducted, we anticipate potential challenges related to political instability, resource constraints, and logistical barriers. To safeguard feasibility, we have built in strategies to prioritize essential components, adapt sequencing, and manage trade-offs without compromising the study’s core objectives.

1. Prioritization of core components*:* The qualitative study and co-design workshops are the nonnegotiable elements essential for this adaptation. The scoping review and pilot are designed to be complementary, but timelines can be adjusted if external constraints arise.
2. Phased adaptation: Activities will not run in parallel across all 3 sites. Instead, learnings from one setting (ie, Pakistan) may inform and streamline work in subsequent sites (ie, Afghanistan).
3. Monitoring*:* Oversight committees, comprising senior team members and local and international partners, will monitor feasibility in real time and make decisions about scaling back or sequencing activities if political or logistical barriers emerge.
4. Contextual safeguards: Given the anticipated instability in Afghanistan, for example, we have planned remote support and contingency arrangements, such as virtual supervision, flexible timelines, and coordination through local leaders, to safeguard study continuity. Engagement with community leaders and CAP members will guide trust-building and help mitigate political or cultural resistance to community mobilization. Regular reviews of risk assessments, clear incident reporting and supervision mechanisms, and contingency plans for alternative data collection will ensure researcher safety and continuity of fieldwork in case of restricted access to certain districts.

#### Expected Outcome of the Adaptation Process

The adaptation process will result in the development of the following:

1. A finalized description of the intervention (including meeting content, the composition of meetings, community meetings, and areas for discussion). The detailed intervention may vary across different sites.
2. A manual for the recruitment and training of staff for PLA implementation in the field.
3. A manual for community mobilizers—a guide to help them deliver the PLA intervention in the field.
4. Completed materials for group meetings, such as flipcharts and other visual aids.

### Phase 3: Piloting of Adapted PLA Materials

We will conduct a focused pilot of 1‐2 meetings at each site to obtain feedback on the co-designed PLA materials and assess initial feasibility and acceptability before moving to implementation. Given resource and time constraints, this will not be a full-scale pilot of the intervention but rather an opportunity to examine key aspects of how the adapted materials and group processes function in real settings.

In rural Pakistan, the adapted PLA materials will be tested in 2 rural clusters (one in Peshawar and one in Swabi), with two community groups (male and female) per cluster. In rural Afghanistan (Kabul) and urban Pakistan (Karachi), the pilot will be conducted in 1 cluster each, with 2 community groups (male and female). Groups will be facilitated by trained male and female community mobilizers under the supervision of senior staff.

Two pilot meetings will be conducted at each site, and the evaluation will focus on 2 core domains:

1. Group dynamics (feasibility of group process): Attendance records (proportion of invitees attending), levels of participation and engagement (observed interaction, interest, and willingness to contribute), and mobilizer reflections on ease of facilitation.
2. Acceptability of PLA materials: Clarity of content, cultural appropriateness, and relevance of flipcharts and participatory activities. These will be assessed through structured field notes, observation checklists, and short exit interviews with a purposive sample of group members.

Data sources will therefore include the following:

1. Attendance record (to capture the feasibility of participation)
2. Observation checklists/field notes (to assess dynamics, participation, and fidelity to facilitation guides)
3. Exit interviews (at least 2 per site) with participants (to capture perceptions of clarity, appropriateness, and suggestions for improvement)
4. Debriefs with community mobilizers and supervisors (to reflect on what was feasible and acceptable to deliver)

Success criteria will be pragmatic: for example, reasonable attendance (≥50% of invited participants attending at least one of two meetings), visible engagement in discussions, and no major concerns about the cultural acceptability of the materials.

Findings will be summarized in a short pilot report structured around these domains. Any revisions to the PLA materials will be informed by participants, mobilizers, and supervisors’ feedback. All modifications will be documented using the FRAME framework, including the rationale for the change [30].

### Patient and Public Involvement

This study has an active program of community engagement for which we have constituted CAPs at each of the project sites. The CAP members represent both men and women from the community who have knowledge and understanding of the local contexts and cultures. The members of the CAPs include individuals living with diabetes, individuals without diabetes, caregivers of diabetic individuals, youth representatives, social workers, community health workers, persons from the seminary or mosque, school, and local women’s representation. CAP members have been involved in designing the topic guides and their translation into the local languages. They have also been involved in identifying participants for the study and will help in disseminating the study findings. Their involvement will improve research quality and help foster a sense of ownership of outputs by the community.

## Results

Under the C4IMPACT, EMPOWER-D was funded and launched in November 2022 by NIHR-United Kingdom (NIHR203248). Following that, the main protocol of the project was developed, which took almost a year. This was followed by detailed planning for adaptation work during which the adaptation protocol was conceived. In early 2024, the team started working simultaneously on scoping reviews and the qualitative study. Topic guides for IDIs and FGDs were prepared and piloted in May 2024. Recruitment and invitations for stakeholders or participants were extended, and IDIs and FGDs began in July 2024 and have been completed in Peshawar, Swabi, and Karachi. As of August 2025, data collection is currently ongoing in Afghanistan. The scoping review has also been completed. Results are anticipated to be published in mid-2026 following the completion of data analysis.

## Discussion

### Framework-Guided Contextual Adaptation

This study hypothesizes that adapting the D-Magic intervention through systematic use of the ADAPT guidance and the FRAME framework will result in contextually relevant, acceptable, and feasible community-based participatory interventions tailored for the prevention and control of T2DM in Afghanistan and Pakistan. By incorporating sociocultural preferences, local resources, and community participation, the adapted intervention is expected to enhance both its effectiveness and sustainability in these fragmented settings.

### Principal Findings

We anticipate that the adaptation process will generate interventions that are culturally appropriate, acceptable to participants, and feasible to implement across 3 diverse sites, building a pathway for future implications in other LMICs. The process is designed such that it maintains the internal logic and core of the original PLA intervention, while allowing for necessary contextual modifications. This balance between fidelity and flexibility is expected to strengthen community ownership and improve the potential for sustained impact on diabetes prevention and control.

### Comparison to Prior Work

Our approach builds upon evidence from the D-Magic trial in Bangladesh, which demonstrated the effectiveness and cost-effectiveness of PLA in reducing diabetes risk factors in a collectivist, low-resource setting. Afghanistan and Pakistan share some social structures and health system challenges, making PLA a potentially transferable and scalable strategy. At the same time, this study contributes to the growing literature on how systematic adaptation frameworks can be applied to EBIs to ensure relevance across diversified populations.

### Strengths and Limitations

A major strength of this study lies in its systematic and participatory approach to adaptation, which focuses on community engagement, ensures transparency in documenting changes, and provides insights into maintaining fidelity. However, we understand that given fragile health systems and potential political instability or security issues, feasibility and fidelity may be affected in ways that are difficult to fully anticipate. As this is a protocol of an adaptation study, the conclusions are based on anticipated rather than observed outcomes.

### Dissemination

The adapted PLA toolkit will be disseminated within the project team for its implementation. Key adaptation findings will also be shared with the CAP members. Our qualitative research findings and the scoping review will be disseminated through high-impact, open-access peer-reviewed journals and at local and international conferences. Reports will also be prepared for policymakers and stakeholders in the countries involved.

### Future Directions

The findings of this study will emphasize the importance of systematic adaptation processes for shaping intervention delivery, effectiveness, and sustainability in implementation research, particularly in resource-constrained and crisis-affected settings. This study will also provide a replicable framework for other EBIs targeting NCDs and related risk factors in LMICs.

### Conclusion

The adaptation of D-Magic for EMPOWER-D is expected to synthesize a culturally grounded intervention that both adheres to its psychosocial origin and responds to the contextual realities of Afghanistan and Pakistan. By applying structured frameworks to guide and document this process, the results are expected to provide insights into how fidelity and contextual relevance can be balanced in complex and diverse, resource-limited settings. The hypothesized findings will inform the design of a definitive trial and contribute to the wider evidence base on the adaptation and implementation of participatory CBIs for NCD prevention and control in LMICs.

## Supplementary material

10.2196/71602 Multimedia Appendix 1 The theory of change logic model for PLA intervention in EMPOWER-D.

10.2196/71602 Multimedia Appendix 2 Topic guide for in-depth interviews with participants from community with unknown diabetes status.

10.2196/71602 Multimedia Appendix 3 Topic guide for in-depth interviews with participants with diabetes.

10.2196/71602 Multimedia Appendix 4 Topic guide for in-depth interviews with carers of people with diabetes.

10.2196/71602 Multimedia Appendix 5 Topic guide for in-depth interviews with health care providers.

10.2196/71602 Multimedia Appendix 6 Topic guide for in-depth interviews with representatives of the community.

10.2196/71602 Multimedia Appendix 7 Focus group discussion topic guide for participants with unknown diabetes status.

10.2196/71602 Multimedia Appendix 8 Focus group discussion topic guide for participants living with diabetes.

## Abbreviations

C4IMPACT: Centre for Improving Mental and Physical Health Together Declarations
CAP: community advisory panel
CBIs: community-based interventions
CHPR: Community Health Participatory Research
D-Magic Trial: Diabetes Mellitus: Action Through Community Groups or mHealth Information for Better Control
EBIs: evidence-based intervention
EMPOWER-D: Engagement of Community Through Participatory Learning and Action for Control and Prevention of Type 2 Diabetes and Its Risk Factors
FGD: focus group discussion
FRAME: The Framework for Reporting Adaptations and Modifications in Evidence-based Interventions
IDI: in-depth interview
LMICs: low- and middle-income countries
NCDs: noncommunicable diseases
PLA: Participatory Learning and Action
PRISMA-ScR: Preferred Reporting Items for Systematic Review and Meta-Analysis Extension for Scoping Reviews
SEM: socioecological model
T2DM: type 2 diabetes mellitus
