# Supplementary material for: Application of the ADAPT Framework to Contextualize a Participatory Learning and Action Community Intervention for the Prevention and Control of Type 2 Diabetes Mellitus in Urban and Rural Settings in Afghanistan and Pakistan: Protocol for Intervention Adaptation
Source: JMIR Res Protoc. 2026 Mar 27;15:e71602. doi: 10.2196/71602 (PMC13026432; doi:10.2196/71602)
Supplement: Multimedia Appendix 3 [file resprot-v15-e71602-s003.docx]

**Topic Guide for In-Depth Interviews with Participants with Diabetes**

| **S. No.** | **Question** | **Probe** |
| --- | --- | --- |
| **Category 1: Knowledge, Experience and Perception of Diabetes**  *First I would like to ask some questions about diabetes* | | |
|  | How did you first come to know of your diabetes? | First symptoms |
|  |  | First encounter with care provider (when / where / type of provider) |
|  | What do you know about diabetes? | Causes |
|  |  | Who/what groups of people are more likely to get diabetes? |
|  |  | Symptoms and physical complications |
|  | How has diabetes affected different aspects of your life? | Social (intra and interpersonal relationships, occupational functioning) |
|  |  | Financial |
|  |  | Mental health |
|  | What do you think are some of the preventive measures for diabetes? | Probe particularly for physical activity and healthy diet as a preventive measure |
|  | In your opinion, what are the different ways of treating diabetes? | Medical treatment – exercise, dietary restrictions, medications, insulin |
|  |  | Alternative treatments |
|  |  | Is diabetes curable with treatment |
|  | How has your experience been living with diabetes? | Understanding and accepting the diagnosis |
|  |  | Handling family and friends’ perceptions and advice |
|  | Would you please explain the various types of treatments and care you sought for diabetes. | Diabetes-related care-seeking pathway – alternative care providers, healthcare providers, access to services, finances, etc. |
|  |  |  |
|  | How do you think diabetes influenced your routine life? | Positive: More conscious of healthy behaviours, taking care of self, valuing life more |
|  |  | Negative: Physical complications, cravings, feels different from other people, mental health |
| **Category 2: Barriers and Facilitators to the Uptake and Maintenance of Healthy Behaviours**  *Now we will talk about some healthy behaviours…* | | |
|  | What do you understand by the term healthy behaviours? Can you give some examples?  *Note to Researcher: Enlist suggested behaviours. FOcus on behaviours affecting health (esp NCDs). Cover diet and exercise in detail.* | Healthy diet – organic foods, vegetables, fruits, water intake, dairy intake |
|  |  | Physical activity |
|  |  | Regular doctor visits |
|  |  | Stress management |
|  | What do you understand by the term unhealthy behaviours? Can you give some examples?  *Note to Researcher: Enlist suggested behaviours. Focus on behaviours affecting health (esp NCDs). Cover smoking and smokeless tobacco in detail.* | Sitting for long hours |
|  |  | Smoking and smokeless tobacco |
|  |  | Other illicit substances |
|  |  | Long screen hours |
|  |  | Poor sleeping hours |
|  |  | Poor eating habits (fast food, binge eating, high salt) |
|  | Are you involved in any of these activities and behaviours?  *Note to Researcher: Explore for both healthy and unhealthy behaviours* | If yes, explore time, extent, investment, and outcome in terms of physical and mental health |
|  |  | If no, explore the reasons behind non-involvement |
|  | Do you exercise / walk regularly? | If yes, explore facilitators and drivers of positive behaviour |
|  |  | If no, explore barriers and hindrances in positive behaviour |
|  | Are there any groups in your community for people with diabetes? | If yes, are you a part of them? Explore their areas of discussion |
|  |  | If no, explore if such groups are needed and will provide support |
|  | In your opinion, are there opportunities for the people to maintain the habit of exercise in your community? | Explore opportunities such as gyms, parks, indoor/outdoor walking spaces, security |
|  |  | Are opportunities the same for all people?  Explore differences in terms of demography – gender, age, socioeconomic status, marital status, and community cultures |
|  |  |  |
|  |  |  |
|  | In your opinion, are there opportunities for the people to maintain the habit of healthy diet in your community? | Explore opportunities such as food affordability, food choices and preferences, access to supermarkets |
|  |  | Are opportunities the same for all people?  Explore differences in terms of demography – gender, age, socioeconomic status, marital status, and community cultures |
|  | In your opinion, are there opportunities for the people to abstain from smoking and smokeless tobacco in your community? | E.g, by creating awareness among people about the dangerous effects of smoking |
|  | In your opinion, what are the factors that can facilitate healthy behaviours for people with diabetes in your community? | Personal factors: e.g: Awareness, health education |
|  |  | Community factors: e.g: Community Based Organisation (CBO) for promotion of health |
|  |  | Health systems factors: e.g: Affordable healthcare; |
|  | In your opinion, what are the factors that can cause hindrance in adapting healthy behaviours for people with diabetes in your community? | Personal factors: e.g: lack of knowledge, poor understanding of diabetes, substance abuse |
|  |  | Community factors: e.g: Poverty; no facilities for exercises, Stigma |
|  |  | Health systems factors: e.g: lack of accessibility and affordability of healthcare, |
| **Category 3: Barriers and Facilitators to the Implementation of a Community-based Intervention**  *Now we will talk about community based interventions…* | | |
|  | What do you understand from the term community-based intervention? | |
|  | Have you ever participated in any community-based education and awareness programs for diabetes and/or any other health-related or other social cause before? If so, can you describe your experience? | Past experiences with PLA and/or any other community-based program |
|  |  | Experiences and learnings from past participation |
|  | Do you think people in your community would be willing to meet together to discuss a social or health related cause? | If not, explore the reasons. |
|  | If we plan a meeting in this area to discuss possible activities and strategies for the management of diabetes, do you think people will come together? | Time of the day and venues for male and female gatherings |
|  |  | Possible demographics of participants |
|  |  | Expected number of people |
|  |  | Motivation to come together |
|  | What do you think are some of the possible challenges or barriers to starting community-based activities for the management of diabetes?  *Note to Researcher: Enlist all suggested challenges and discuss the causes and consequences of challenges, and ideas to overcome each one of them* | |
