# Supplementary material for: Application of the ADAPT Framework to Contextualize a Participatory Learning and Action Community Intervention for the Prevention and Control of Type 2 Diabetes Mellitus in Urban and Rural Settings in Afghanistan and Pakistan: Protocol for Intervention Adaptation
Source: JMIR Res Protoc. 2026 Mar 27;15:e71602. doi: 10.2196/71602 (PMC13026432; doi:10.2196/71602)
Supplement: Multimedia Appendix 7 [file resprot-v15-e71602-s007.docx]

**Focus Group Discussion Topic Guide for Participants without Diabetes**

| **S. No.** | **Question** | **Probe** |
| --- | --- | --- |
|  | Please share with us your name and something you love to do in your free time. | Ice breaking question |
| **Category 1: Knowledge, Experience and Perception of Diabetes**  *We would like to know a bit about your understanding of diabetes* and your experience of the disease. | | |
|  | What do you people know about diabetes? | Causes |
|  |  | Who/what groups of people are more likely to get diabetes? |
|  |  | Symptoms and complications |
|  | How can diabetes affect different aspects of peoples’ life? | Positive: More conscious of healthy behaviors, taking care of self, valuing life more |
|  |  | Negative: Physical complications, cravings, feels different from other people, mental health |
| **Category 2: Barriers and Facilitators to the Uptake and Maintenance of Healthy Behaviors**  *We have some questions about barriers and facilitators to health behavior***.** We will discuss factors and activities which promote good health and activities which lead to unhealthy life. | | |
|  | What do you people understand by the term healthy behaviors?  *Note to Researcher: Enlist suggested behaviors. Cover diet, exercise, and stress management in detail.* | Healthy diet – organic foods, vegetables, fruits, water intake, dairy intake |
|  |  | Physical activity |
|  |  | Emotional wellbeing, stress management |
|  | What do you understand by the term unhealthy behaviors? Can you give some examples  *Note to Researcher: Enlist suggested behaviors. Focus on behviours affecting health (esp NCDs). Cover smoking and smokeless tobacco in detail.* | Sitting for long hours |
|  |  | Smoking and smokeless tobacco |
|  |  | Other illicit substances |
|  |  | Long screen hours |
|  |  | Poor sleeping hours |
|  |  | Poor eating habits (fast food, binge eating, high salt) |
|  | What kind of behaviors do you think people with diabetes are you involved in? | Explore time, extent, investment, and outcome in terms of physical and mental health |
|  | Do you think there is a role of healthy diet and physical activity in managing diabetes? | Explore understanding of positive effects of physical activity and dietary restrictions on blood sugar, quality of life, good mental health outcomes, and prevention of complications |
|  | In your opinion, what are the factors that can facilitate healthy behaviors for people with diabetes in your community? | Personal factors: e.g: |
|  |  | Community factors: e.g: |
|  |  | Health systems factors: e.g: |
|  | In your opinion, what are the factors that can cause hindrance in adapting healthy behaviors for people with diabetes in your community? | Personal factors: e.g: |
|  |  | Community factors: e.g: |
|  |  | Health systems factors: e.g: |
| **Category 3: Barriers and Facilitators to the Implementation of a Community-based Intervention**  *We would like to ask a few questions about community-based interventions*, i.e., how communities can work collectively to promote healthy behavior for the management and control of diabetes. | | |
|  | What do you understand from the term community-based intervention? | |
|  | Have you ever participated in any community-based education and awareness programs for diabetes and/or any other disease before? If so, can you describe your experience? | Past experiences with any other community-based program |
|  |  | Experiences and learnings from past participation |
|  | Do you think people in your community would be willing to meet together to discuss a social or health related cause? | If not, explore the reasons. |
|  | What are some of the possible challenges or barriers to starting community-based activities for the management of diabetes?  *NR: Enlist all suggested challenges and discuss the causes and consequences of challenges, and ideas to overcome each one of them* | |
